# CRISPRseek: A Bioconductor Package to Identify Target-Specific Guide RNAs for CRISPR-Cas9 Genome-Editing Systems

**DOI:** 10.1371/journal.pone.0108424

**Published:** 2014-09-23

**Authors:** Lihua J. Zhu, Benjamin R. Holmes, Neil Aronin, Michael H. Brodsky

**Affiliations:** 1 Program in Gene Function and Expression and Program in Molecular Medicine, University of Massachusetts Medical School, Worcester, MA, United States of America; 2 Program in Bioinformatics and Integrative Biology, University of Massachusetts Medical School, Worcester, MA, United States of America; 3 Broad Institute of MIT and Harvard, McGovern Institute for Brain Research at MIT, Departments of Brain and Cognitive Sciences and Biological Engineering, MIT, Cambridge, MA, United States of America; 4 RNA Therapeutics Institute and Department of Medicine, University of Massachusetts Medical School, Worcester, MA, United States of America; University of Florida, United States of America

## Abstract

CRISPR-Cas systems are a diverse family of RNA-protein complexes in bacteria that target foreign DNA sequences for cleavage. Derivatives of these complexes have been engineered to cleave specific target sequences depending on the sequence of a CRISPR-derived guide RNA (gRNA) and the source of the Cas9 protein. Important considerations for the design of gRNAs are to maximize aimed activity at the desired target site while minimizing off-target cleavage. Because of the rapid advances in the understanding of existing CRISPR-Cas9-derived RNA-guided nucleases and the development of novel RNA-guided nuclease systems, it is critical to have computational tools that can accommodate a wide range of different parameters for the design of target-specific RNA-guided nuclease systems. We have developed *CRISPRseek*, a highly flexible, open source software package to identify gRNAs that target a given input sequence while minimizing off-target cleavage at other sites within any selected genome. *CRISPRseek* will identify potential gRNAs that target a sequence of interest for CRISPR-Cas9 systems from different bacterial species and generate a cleavage score for potential off-target sequences utilizing published or user-supplied weight matrices with position-specific mismatch penalty scores. Identified gRNAs may be further filtered to only include those that occur in paired orientations for increased specificity and/or those that overlap restriction enzyme sites. For applications where gRNAs are desired to discriminate between two related sequences, *CRISPRseek* can rank gRNAs based on the difference between predicted cleavage scores in each input sequence. *CRISPRseek* is implemented as a Bioconductor package within the R statistical programming environment, allowing it to be incorporated into computational pipelines to automate the design of gRNAs for target sequences identified in a wide variety of genome-wide analyses. *CRISPRseek* is available under the GNU General Public Licence v3.0 at http://www.bioconductor.org.

## Introduction

The clustered, regularly interspaced, short palindromic repeats (CRISPR) found in many prokaryotes encode RNAs that act together with CRISPR-associated proteins (Cas) and a tracRNA to function as an adaptive immune system to detect and cleave foreign DNA sequences [1], [2], [3]. Modified versions of CRISPR-Cas systems have been developed that use a CRISPR-derived guide RNA (gRNA) sequence to direct the nuclease activity of Cas9 proteins to specific targets within the genome [4]. gRNA-directed Cas9 systems can create targeted genetic changes in human stem cells and in model and non-model organisms [5], [6], [7], [8], [9], [10], [11], [12]. These nucleases create double strand DNA breaks that can result in a variety of genome modifications including short insertion/deletions (indels) via non-homologous end-joining or specific sequence changes introduced via homology-directed repair with a DNA donor molecule [13]. Other useful derivatives of gRNA-Cas9 complexes include nickases, which only cleave one DNA strand and can be used to increase specificity in paired configurations, and gene expression regulators, which lack any DNA cleavage activity, but can increase or decrease gene transcription by recruiting additional effector domains [4], [13].

**Figure 1:**
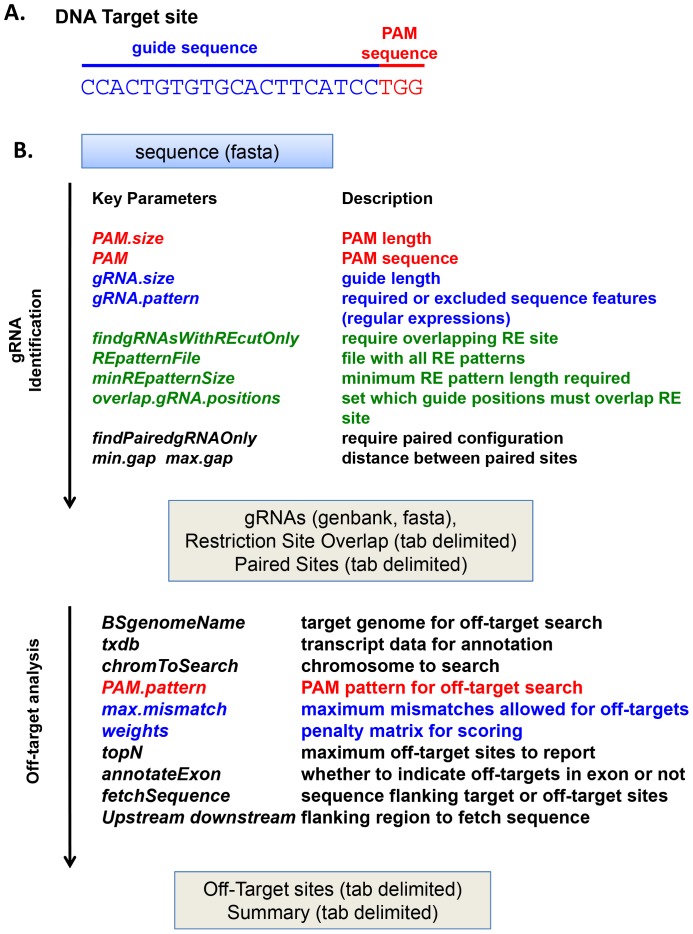
Major parameters to identify gRNA-Cas9 target and off-target sites with the *offTargetAnalysis* function of *CRISPRseek*. **A.** Target sequences for CRISPR-Cas9-derived nucleases are composed of two components, the guide sequence (red) that matches the variable region in the guide RNA and an associated PAM sequence that is recognized by the Cas9 protein. The sequence shown is a possible target sequence for a nuclease based on the *S. pyogenes* CRISPR-Cas9 system, which has a 20 base pair guide sequence and a PAM sequence of NGG or NAG. **B.** *offTargetAnalysis* first identifies candidate guide sequences in an input sequence. Some of the possible parameters to identify these sequences are indicated, including parameters for the guide sequence, the adjacent PAM sequence and other features such as overlapping restriction enzyme sites. Next, potential off-target sites for the identified guide sequences are identified in a specified genome. A variety of parameters can be adjusted to determine the criteria used to identify and score off-target sites.

gRNA-Cas9 complexes recognize specific target sequences composed of two components (Figure 1A). The guide sequence is typically identical to a variable region in the gRNA. Directly adjacent to the guide sequence is the “protospacer adjacent motif” (PAM) sequence, which is a short sequence recognized by the Cas9 protein [14]. In the most widely used CRISPR-Cas9 system derived from *S. pyogenes* [4], [13], the guide sequence is typically a variable region of 20 bases while the preferred PAM sequence is NGG (with lower activity at NAG). One potential limitation for CRISPR-Cas9 systems is that they can cleave at sequences that do not precisely match the sequence targeted by the gRNA [15], [16], [17], [18]. Thus, an important consideration for the design of CRISPR-Cas9 systems is the selection of gRNAs with low rates of off-target cleavage. Early studies suggested that only the 12 bases in the guide sequence closest to the PAM sequence contribute to specificity [6]. A more recent analysis used extensive experimental data to model cleavage likelihood for off-targets sites based on the location, the number and arrangement of mismatches [17], and derived a mismatch penalty matrix and scoring scheme that were used for a web application to select gRNAs with minimum off-target cleavage at http://crispr.mit.edu [13]. Off-target rates can be further reduced using strategies that require binding of two gRNA-Cas9 complexes, such as paired Cas9 nickases [15], [18], [19] or dimers of a nuclease-dead Cas9 fused to the FokI nuclease [20].

To facilitate the use of different classes of CRISPR-Cas9 systems by a wide range of investigators, there is a need for platform-independent and genome-independent tools to identify gRNAs that cleave single or paired target sites for various CRISPR-Cas9 systems and to perform off-target analysis. Two previously described tools can be run locally with different genomes [21], [22], but do not incorporate available experimental information about the relative contributions of different numbers, positions and arrangements of mismtaches on the cleavage of off-target sites [17]. A more sophisticated tool utilizing this information has been made available as a webserver [13], which is easy to use, but restricted to the search parameters and genomes provided by the website. Here we describe an open source Bioconductor package, *CRISPRseek*, to facilitate design of target-specific gRNAs for any CRISPR-Cas9 system with a characterized PAM sequence and off-target analysis with any available genome sequence using experimentally-derived off-target scoring schemes [17]. In addition, we provide the functionality to score target sites in two related input sequences in order to identify gRNAs that are, for example, specific for one target and not the other. Furthermore, users have the flexibility to supply their own mismatch penalty matrix, allow variations in PAM sequences, specify specific patterns for guide sequences and modify other search criteria; thus, these features will allow target site search parameters to continue to be modified as additional classes of CRISPR-Cas-derived systems are characterized and new experimental information on off-target activity becomes available.

## Materials and Methods

*CRISPRseek* implements a common gRNA design and off-target analysis workflow for CRISPR-Cas9-derived systems in R, a system for statistical computation and graphics [23], [24]. To promote component reuse and compatibility among Bioconductor packages, *CRISPRseek* utilizes the *Biostrings* package to represent sequences and find matches. To efficiently determine whether off-target sequences overlap with any exon, *GenomicRanges* is used to represent genomic coordinates. *BSgenome* is employed to retrieve flanking sequences of off-targets. *CRISPRseek* leverages various sources of genomes as *BSgenome* object and annotations such as *TransciptDb* object in Bioconductor. An experimentally-determined position specific mismatch penalty matrix and scoring scheme were used for predicting cleavage score for potential off-targets [17]. *CRISPRseek* emphasizes flexibility and ease of use. gRNA identification and filtering, and off-target searching, scoring, and annotation are integrated into one workflow function *offtargetAnalysis*. Once the package is loaded and parameters are set, one line of code (*offtargetAnalysis*) can perform all the analysis by calling various helper functions. A second workflow function *compare2sequences* is for identifying gRNAs that target one of two related sequences or both sequences.

An installation guide and additional generic use cases for *CRISPRseek* are described in the Supplemental method file and in the Vignettes and Manual provided with the R package (available at Bioconductor.org).

## Results and Discussion

### gRNA Identification

*CRISPRseek* identifies and evaluates candidate gRNAs for CRISPR-Cas9-derived nucleases to target a given input sequence using various constraints and also reports and ranks potential off-target sequences for each gRNAs. The *offTargetAnalysis* function can be used to perform customized searches for gRNAs to target a given sequence and for off-target sites in a specified genome (Figure 1B). A genomic DNA sequence of interest is inputted as a fasta formatted file. A variety of parameters can be adjusted to specify the on-target and off-target sites identified in this search. The identified gRNA sequences can be exported in a variety of formats, including a genbank file format to allow visualization in many sequence analysis programs. The default parameter settings for the *offTargetAnalysis* function are:

*offTargetAnalysis (inputFilePath, format = “fasta”, findgRNAs = TRUE, exportAllgRNAs = c(“all”, “fasta”, “genbank”, “no”), findgRNAsWithREcutOnly = TRUE, REpatternFile, minREpatternSize = 6, overlap.gRNA.positions = c(17, 18), findPairedgRNAOnly = TRUE, min.gap = 0, max.gap = 20, gRNA.name.prefix = “gRNA”, PAM.size = 3, gRNA.size = 20, PAM = “NGG”, BSgenomeName, chromToSearch = “all”, max.mismatch = 4, PAM.pattern = “N[A|G]G$”, gRNA.pattern = “”,*
*min.score = 0.5, topN = 100, topN.OfftargetTotalScore = 10, annotateExon = TRUE,*
*txdb, outputDir, fetchSequence = TRUE, upstream = 200, downstream = 200,*
*weights = c(0, 0, 0.014, 0, 0, 0.395, 0.317, 0, 0.389, 0.079, 0.445, 0.508, 0.613, 0.851, 0.732, 0.828, 0.615, 0.804, 0.685, 0.583), overwrite = FALSE).*

Target sites are composed of two components, the guide sequence, which is determined by the variable region of the gRNA, and the PAM sequence, which is recognized by the Cas9 protein (Figure 1A). If a specific set of target sites are already identified, they can be entered as a multi-sequence fasta file with the *findgRNAs* command set to FALSE. The default constraints for a target site search is set for sites recognized by the widely used Cas9 nuclease from *S. pyogenes,* composed of a 20 base guide sequence and a 3 base preferred PAM sequence (NGG). The PAM length and sequence can be altered to use the preferred sequences for Cas9 systems from other species [25], [26] using the *PAM* and *PAM.size* commands. The *gRNA.size* command can be altered to adjust the identified guide sequence length for Cas9 systems from other species that utilize different gRNA lengths or for truncated gRNAs [27], which may provide greater specificity. The gRNA sequence can also be constrained to optimize the efficiency of CRISPR-Cas9 systems by setting *gRNA.pattern* to require or exclude specific features within the target site. For example, synthesis of gRNAs *in vivo* from host U6 promoters is more efficient if the first base is guanine and gRNA synthesis *in vitro* using T7 promoters is most efficient when the first two bases are GG [13]; these features can be specified by setting *gRNA.pattern* = *“∧G”* and *“∧GG”* respectively (Figure 1B). Another example is that five consecutive uracils in any position of a gRNA will affect transcripion elongation by RNA polymerase III. To avoid premature termination during gRNA synthesis using U6 promoter, we can set *gRNA.pattern* = *“∧(?:(?!T{5,}).)+$”.* In addition, some studies have identified sequence features that broadly correlate with lower nuclease cleavage activity, such as uracil in the last 4 positions of the guide sequence [28]; to avoid uracil in these positions, we can specify *gRNA.pattern* = *“[ACG]{4,}.{3}$”.*

*CRISPRseek* can also filter candidate target sites based on other features. Appropriately orientated and spaced pairs of target sites can be used with nickase-derivatives of CRISPR-Cas9 systems to increase cleavage specificity [15], [19]. Reported target sites can be filtered such that only paired sites are recovered. While the spacing for these sites has been described for CRISPR-Cas9 systems derived from *S. pyogenes*, it is possible that other spacing constraints is required for CRISPR-Cas9 systems from other species or for novel dimeric nucleases such as CRISPR-RNA guided FokI nucleases [20]. The command *findPairedgRNAOnly* = TRUE restricts the output to paired sites while the *min.gap* and *max.gap* commands allows alternative spacings to be set for paired target sites. An additional feature that can be useful for monitoring nuclease cleavage events is the presence of RE cut sites that overlap the gRNA-Cas9 cleavage site; The *findgRNAsWithREcutOnly, REpatternFile, minREpatternSize, overlap.gRNA.positions* can be adjusted to require that the Cas9 cleavage site overlaps with RE sites with a particular length recognition site. A list of enzymes and their recognition sites from a commercial supplier is provided with the package.

### Identification of potential off-target sequences

An additional criteria for selecting gRNAs is to minimize the likelihood that other sites in the host genome are cleaved by the nuclease (Figure 1B). *CRISPRseek* takes advantage of the Bioconductor *BSgenome* package, which provides a large number of pre-formated genomes for well-studied organisms and tools to format additional genome sequences. *offTargetAnalysis* will search all chromosomes of the specified genome in the default setting of *chromToSearch* (“all”). It can be adjusted to skip the off-target search step (“”) or to search individual chromosomes if, for example, a specific gene cluster is of particular interest (“chr1”). The default search of genomic sequences for potential off-target sites allows maximum of 4 mismatches, which can be modified by users with the *max.mismatch* parameter. If desired, the PAM sequence for off-target searches set by the *PAM.pattern* parameter can be different from the PAM sequence used for target identification to allow variants of PAM sequences to be scanned as potential off-target sites. For example, Cas9 from *S. pyogenes* recognizes NGG PAM sequences and, to a lesser extent, NAG [17]. To increase the specificity of the identified gRNAs, we set *PAM* = “NGG” for candidate gRNA identification while setting *PAM.pattern* = “N[A|G]G” for potential off-target finding.

Prediction of relative off-target cleavage rates is based on previously described approach in which an experimentally-determined penalty matrix is used to score the relative effect of single and multiple mismatches in the off-target sequence [17]. However, the *weights* parameter can be readily adjusted to incorporate alternative matrices for *S. pyogenes* Cas9 or different penalty matrices determined for either Cas9 from other species or for gRNAs with truncated guide sequences [26], [27]. The predicted off-target cleavage score ranges from a maximum of 100 for sites predicted to cleave as well as the intended target to 0 for sites that do not resemble the target sequence. The total number of off-target sequences reported can be adjusted by setting the maximum number of mismatches allowed in sequences, by setting a minimum score for reported off-targets, and by directly setting the number of off-targets reported.

Off-target analysis results are reported as four tab-delimited files: *Summary.xls* (target sequence summaries), *REcutDetails.xls* (RE cut sites overlapping the nuclease cut site), and *pairedSpacers.xls* (potential paired spacers), and *Off-targetAnalysis.xls* (off-target sequences and scores). Recovered gRNAs are also reported in genbank and fasta formats to readily allow further analysis or visualization with other software.

### Compare two sequences

**Figure 2:**
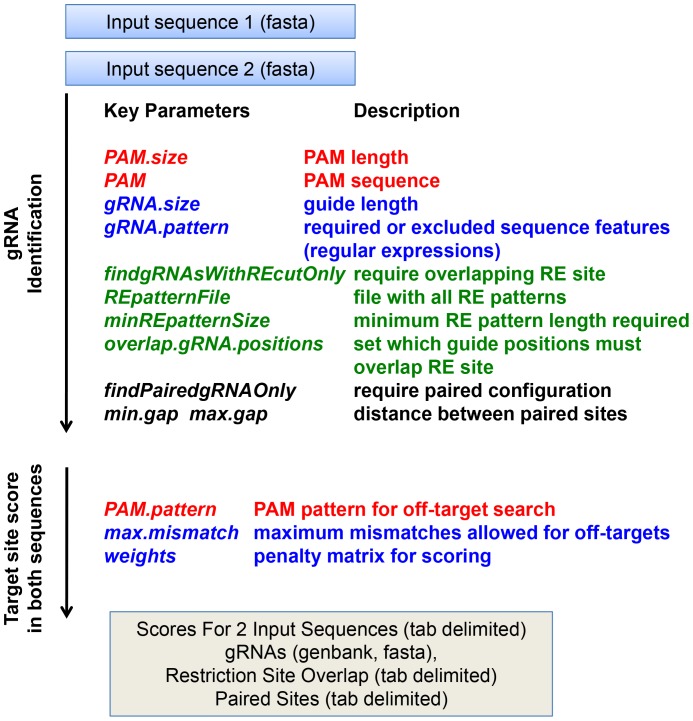
Major parameters to identify gRNA-Cas9 target sites that are shared or distinct in two related sequences with the *compare2sequences* function of *CRISPRseek*. * compare2sequences* first identifies target sites in two input sequences using many of the same parameters available for the *offTargetSequence* function. Next, cleavage scores are determined for each target site in both target sequences.

In a variety of experimental contexts, it may be useful to identify target sites that are specific for one of two related sequences. For example, if a target gene is a member of a gene family, it may be desirable to specifically search for target sites that are not present in other members of that family. Also, if a site-specific mutation in a target gene is being introduced by homologous recombination, the investigator may wish to target the original sequence, but not the sequence to be introduced. If polymorphisms are present in a gene, the investigator may wish to target only one of the two alleles. Alternatively, it may be desirable to identify gRNAs that are not affected by those sequence differences so that both alleles can be targeted with a single nuclease. The *compare2Sequences* function facilitates the identification of gRNAs of CRISPR-Cas9-derived systems that target either one or both input sequences (Figure 2). Both sequences are inputted in fasta format. The default settings called by *compare2Sequence* are:

*compare2Sequences (inputFile1Path, inputFile2Path, format = “fasta”,*
*findgRNAsWithREcutOnly = FALSE, REpatternFile, minREpatternSize = 6,*
*overlap.gRNA.positions = c(17, 18), findPairedgRNAOnly = FALSE,*
*min.gap = 0, max.gap = 20, gRNA.name.prefix = “gRNA”, PAM.size = 3,*
*gRNA.size = 20, PAM = “NGG”, PAM.pattern = “N[A|G]G$”, outputDir,*
*weights = c(0, 0, 0.014, 0, 0, 0.395, 0.317, 0, 0.389, 0.079, 0.445, 0.508, 0.613, 0.851, 0.732, 0.828, 0.615, 0.804, 0.685, 0.583), overwrite = FALSE).*

These settings are largely similar to those for the *offTargetAnalysis* function, except that two input sequences are provided and no genome-wide off-target analysis is performed. Instead, the predicted cleavage scores are calculated for target sites in both sequences. Sites with the desired properties (either present in both or specific to one) can then be further characterized via the *offTargetAnalysis* command.

### Example Analysis

**Figure 3:**
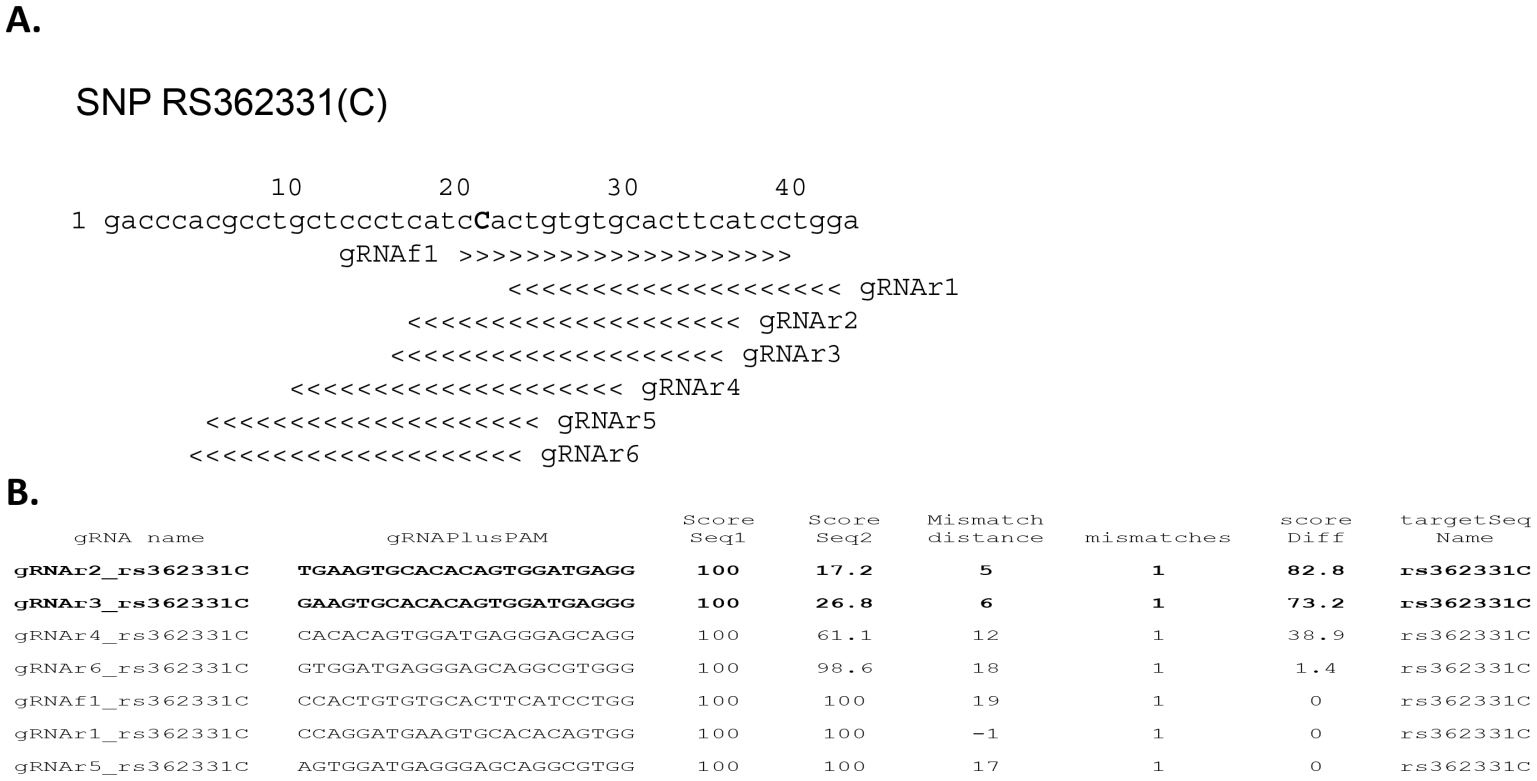
An example of using *compare2sequences* to identify gRNAs that target for the C allele of a SNP in the human *huntingtin* gene. **A.** Sequences for both alleles of the SNP RS362331 were used as inputs for *compare2sequences*. The input sequence used for the C allele is shown while the T allele is identical except at the position in bold. Positions of potential gRNA sequences for the C allele are visualized by inporting the genbank format output from *compare2sequences* into the “A plasmid Editor” or ApE sequence analysis program and displaying a graphical map. **B.** A partial output of gRNAs and their cleavage scores in each input sequence is shown. gRNAs are numbered based on their orientation (f, forward. r, reverse complement), position and input sequence name. Target site sequences include the gRNA sequence and the adjacent PAM sequence. Cleavage scores are calculated for the gRNA in both input sequences and the name and position of each mismatch relative to the PAM sequence is shown. The cleavage score difference indicates the likehood that a gRNA with a target site in one input sequence will also cleave the other input sequence. A score difference of “0” indicates that the gRNAs are predicted to cleave both sequences with equal efficiency; “100” indicates that the gRNAs are predicted to be specific for just one sequence. The gRNAs in bold are candidates to show greater activity for the C allele compared with the T allele.

In Figure 3 and Files S1–S7, we demonstrate the results from a specific application of *CRISPRseek* to select gRNAs that target allele-specific sites in the human Huntington’s disease gene (*HTT*). Expansion of CAG repeats in one copy of *HTT* can result in adult onset neurodegeneration. Because *HTT* is an essential gene, nucleases cannot be used that inactivate both alleles [29]. Therefore, to identify nuclease target sites that are allele-specific, we searched for sites that overlap a single nucleotide polymorphism (SNP), RS362331 [30] located in a coding exon of *HTT*. Two sequences that differ only at the polymorphism site were used as inputs for *compare2sequences* (Figure 3A, Files S1–S2). Six target sites were identified for the sequence with the T allele and seven for the C allele (File S3). The sequence and location of these target sequences is outputted in Fasta and genbank formats (Files S4–S6), allowing ready production of figures indicating their positions (Figure 3A); in the example shown, the genbank file containing seven potential target sites for the C allele was imported into the sequence analysis program ApE (http://en.wikipedia.org/wiki/Ape) and gRNA positions were visualized using the Graphic Display function. For each target site, a cleavage score was predicted for each input sequence where the maximum cleavage score is 100 (Figure 3B, File S3). Two target sites for the C (gRNAr2 and gRNAr3) alleles had scores under 30 for the other allele (Figure 3B) due to a mismatch near the CRISPR-Cas9 cleavage site, which is three bases from the PAM sequence. These target sites represent possible targets for allele-specific gRNA-Cas9 nucleases. Conversely, they may be poor choices for nucleases intended to efficiently cleave both alleles.

Subsequently, the top two potential gRNAs targeting the genomic DNA sequence flanking SNP RS362331 with the C allele were used for off-target analysis with *offTargetAnalysis* function (File S6). The top scoring hit typically corresponds to the target sequence in the reference genome. In this case, the reference genome has the other allele, so most of the top matches have a single mismatch. The top off-target sites have 2–3 mismatches. The number and position of these mismatches are used to generate an off-target cleavage score and the summary file that reports the sum of the top 5 and top 10 off-target sites, which are the most likely off-target sites to be cleaved in a targeting experiment. The combined off-target scores for the top 10 best matches in the genome for gRNAr2 and gRNAr3 are 13.1 and 15.9 (File S6, sheet 1), indicating that these sites represent candidate targets to maximize allele-specificity and minimize off-target cleavage. Additional information describing all analyzed off-target sites (including genomic location, whether the site is exonic, flanking genomic sequences for primer design, and potential overlapping RE sites) are also included in this file.

## Concluding Remarks

The rapidly changing available information for existing CRISPR-Cas9 derived systems and ongoing development of these nucleases from new species creates a need for software that can flexibly incorportate the latest information into the identification and evaluation of gRNAs and off-target sites. We have developed the Bioconductor package *CRISPRseek* to facilitate design of target-specific gRNAs for effective application of the CRISPR-Cas9 system. *CRISPRseek* offers the ability/flexibility to search any species with available sequence by utilizing *BSgenome* function and to use target sequence preferences for CRISPR-Cas9 systems from any characterized bacterial species. It also provides tools to look for sites that are arranged in pairs, that overlap RE sites or that are present in only one of two related sequences. The inclusion of *CRISPRseek* in Bioconductor also allows it to be incorporated in novel workflows to design gRNAs to target sequences identified in genome-wide studies or to further characterize the on or off-target sequences from the *CRISPRseek* output.

## Supporting Information

File S1 Fasta file with sequence for C allele of human single nucleotide polymorphism RS362331.
(FA) Click here for additional data file.

File S2 Fasta file with sequence for T allele of human single nucleotide polymorphism RS362331.
(FA) Click here for additional data file.

File S3 Tab delimited file outputs from *compare2sequences* function of *CRISPRseek* using files S1 and S2 as inputs.
(XLSX)

File S4 Genbank formated files with gRNA identified by *compare2sequences* function of *CRISPRseek* using files S1 and S2 as inputs.
(GBK)

File S5 Genbank formated files with gRNA identified by *compare2sequences* function of *CRISPRseek* using files S1 and S2 as inputs.
(GBK)

File S6 Fasta formatted file of gRNAs identified for the C allele by *compare2sequences* function of *CRISPRseek* using files S1 and S2 as inputs.
(FA) Click here for additional data file.

File S7 R commands used to identify gRNAs that target the C allele of a SNP from the Huntington’s Disease locus.
(DOCX)

File S8 A basic introduction to installing Bioconductor, CRISPRseek, and different data packages required for gRNA identification by CRISPRseek.
(DOCX)
