# Supplementary material for: CRISPRseek: A Bioconductor Package to Identify Target-Specific Guide RNAs for CRISPR-Cas9 Genome-Editing Systems
Source: PLoS One. 2014 Sep 23;9(9):e108424. doi: 10.1371/journal.pone.0108424 (PMC4172692; doi:10.1371/journal.pone.0108424)
Supplement: File S7 — R commands used to identify gRNAs that target the C allele of a SNP from the Huntington’s Disease locus. (DOCX) [file pone.0108424.s007.docx]

This file describes the commands used in the example analysis described in Results section. For the *compare2sequences* analysis, the user must have already installed R and *CRISPRseek* packages. For the *offTargetAnalysis*, the user must also install the human genome *("BSgenome.Hsapiens.UCSC.hg19")* and the human genome transcript annotation *("TxDb.Hsapiens.UCSC.hg19.knownGene")* packages. A more basic guide to install *CRISPRseek* and related packages is provided in Supplemental File 8.

**1. Use *compare2sequences* to identify and compare gRNAs for a single nucleotide polymorphism in the human Huntingtin gene.** Supplemental Files 1 and 2 contain fasta files for each sequence. Note that the working directory (wd) must be set to the folder containing these files.

*library(CRISPRseek)*

*outputDir <- getwd()*

*inputFile1Path <- "SupplementalFile1.fa"*

*inputFile2Path <- "SupplementalFile2.fa"*

*REpatternFile <- system.file("extdata", "NEBenzymes.fa", package = "CRISPRseek")*

*seqs <- compare2Sequences(inputFile1Path, inputFile2Path, outputDir = outputDir , REpatternFile = REpatternFile, overwrite = TRUE)*

*seqs*

**2. Use *offTargetAnalysis* to identify possible off-target sites for gRNAs identified in the *compare2sequences* analysis.**

*library(CRISPRseek)*

*library("BSgenome.Hsapiens.UCSC.hg19")*

*library(TxDb.Hsapiens.UCSC.hg19.knownGene)*

*outputDir <- getwd()*

*inputFilePath <- "* *SupplementalFile7.fa*

*REpatternFile <- system.file("extdata", "NEBenzymes.fa", package = "CRISPRseek")*

*offTargetAnalysis(inputFilePath, findgRNAs = FALSE, findgRNAsWithREcutOnly = FALSE, REpatternFile = REpatternFile, findPairedgRNAOnly = FALSE, BSgenomeName = Hsapiens, txdb = TxDb.Hsapiens.UCSC.hg19.knownGene, max.mismatch = 3, chromToSearch = "all", outputDir = outputDir, overwrite = TRUE)*
