# Supplementary material for: CRISPRseek: A Bioconductor Package to Identify Target-Specific Guide RNAs for CRISPR-Cas9 Genome-Editing Systems
Source: PLoS One. 2014 Sep 23;9(9):e108424. doi: 10.1371/journal.pone.0108424 (PMC4172692; doi:10.1371/journal.pone.0108424)
Supplement: File S8 — A basic introduction to installing Bioconductor, CRISPRseek, and different data packages required for gRNA identification by CRISPRseek. (DOCX) [file pone.0108424.s008.docx]

**Installation and use of CRISPRseek (intended for new users of R and Bioconductor).**

CRISPRseek is a software package that operates within the R programming environment and has been accepted as a Bioconductor package. Bioconductor is an open source environment for bioinformatics software written in R. It has semi-annual releases containing hundreds of software packages for bioinformatics and genome analysis.

We describe here how to install it using the most current stable release of R. We attempt to make these installation instructions clear for biologists with little or no experience using command line for informatics. These instructions use installation on Mac OS as an example, but R and CRISPRseek run on other operating systems such as Windows and Linux. For further help see the CRISPRseek user manual and the Bioconductor.org website.

**Step 1: Install R and Bioconductor.** Instructions are at http://www.bioconductor.org/install/. To install R, go to <http://www.r-project.org>, select the closest mirror site (e.g. <http://watson.nci.nih.gov/cran_mirror/>), click on [Download R for (Mac) OS X](http://watson.nci.nih.gov/cran_mirror/bin/macosx/) and then select the [latest version](http://watson.nci.nih.gov/cran_mirror/bin/macosx/R-latest.pkg) to download. Precompiled binary distributions are also available for Windows and Linux. Additional steps may be required for some operating systems. Next, to install the core set of Bioconductor packages, open the R application and type these commands after the cursor in the R Console:

*source("http://bioconductor.org/biocLite.R")*

*biocLite()*

The biocLite command is frequently used to download/install additional Bioconductor data or software packages into R.

**Step 2. Install CRISPRseek package and get documentation.**

To install the package:

*biocLite(CRISPRseek)*

To load the package:

*library(CRISPRseek)*

To open a window with documentation about the package:

*help(CRISPRseek)*

To open a window with an example of the most commonly used function:

*help(offTargetAnalysis)*

To view additional documentation with examples:

*browseVignettes("CRISPRseek")*

**Step 3: Install genome sequence and annotation packages for off-target analysis.** To search for off-target sites and annotate if they are in exons, data packages containing the relevant genome sequence and transcript annotations must be installed and loaded. The example off-target analysis requires the human genome package. Install the human genome with the following command:

*source("http://bioconductor.org/biocLite.R")*

*biocLite("BSgenome.Hsapiens.UCSC.hg19")*

(Large genomes may take awhile to download.) Next, install the genome annotation file:

*biocLite("TxDb.Hsapiens.UCSC.hg19.knownGene")*

Many other genomes can be found at bioconductor.org by searching for BSgenome AND the species name. Annotations can be found by searching for TxDb AND the species name**.** Commands to install some common BSgenome and TxDb annotation packages are:

*source("http://*[*bioconductor.org/biocLite.R*](http://bioconductor.org/biocLite.R)*")*

*biocLite("BSgenome.Hsapiens.UCSC.hg19")*

*biocLite("BSgenome.Mmusculus.UCSC.mm10")*

*biocLite("BSgenome.Rnorvegicus.UCSC.rn5")*

*biocLite("BSgenome.Drerio.UCSC.danRer7")*

*biocLite("BSgenome.Dmelanogaster.UCSC.dm3")*

*biocLite("BSgenome.Celegans.UCSC.ce6")*

*biocLite("TxDb.Hsapiens.UCSC.hg19.knownGene")*

*biocLite("TxDb.Mmusculus.UCSC.mm10.knownGene")*

*biocLite("TxDb.Rnorvegicus.UCSC.rn5.refGene")*

*biocLite("TxDb.Dmelanogaster.UCSC.dm3.ensGene")*

*biocLite("TxDb.Celegans.UCSC.ce6.ensGene")*

If the genome of interest does not have the annotation file, you can still run CRISPRseek without it by setting *annotateExon = FALSE*. You can make your own TxDb file with makeTranscriptDbFromUCSC in the GenomicFeatures package. Here is an example to create a Zebrafish TranscriptDb using either Ensembl or Entrez genes.

*biocLite("GenomicFeatures")*

*library(GenomicFeatures)*

*txdb <- makeTranscriptDbFromUCSC("danRer7", "ensGene")*

or

*txdb <- makeTranscriptDbFromUCSC("danRer7", "refGene")*

(For both gene models, you can ignore warnings that the cds cumulative length is not a multiple of 3 for many transcripts.)

**Step 4. Save a fasta format file with your target sequence in the working directory.** Make sure to use the correct format for fasta files. You can use an application such as textedit to construct it in a Plain Text format and save the file with “.fa” as the suffix. Put this file in the folder designed as your working directory. Commands to identify or change the working directory are under the “Misc” drop down menu. Your output files will be in this directory as well.

Large sequences will have many candidate gRNAs, potentially resulting in long run times for the genome-wide off-target search. To limit run times, keep the target sequence short or first identify gRNAs and then select a subset for off-target analysis.

(Technical note: different operating systems may use different character codes to terminate lines in text files, which may interfere with the interpretation of the sequence by R. Windows uses a pair of CR and LF characters to terminate lines. UNIX (Including Linux and FreeBSD) uses an LF character only. Some MAC files use a single CR character for line breaks. If your MAC file is not recognized by CRISPRseek, you may need to change CR to LF. If so, open the terminal and run the following commands:

*cd* ***directory***

*more* ***X****.fa | tr "\r" "\n" >* ***Y****.fa*

Where **directory** = the directory where file **X**.fa resides and **Y**.fa = the revised file name.)

**Step 5. Design guide RNAs (gRNAs) with CRISPRseek.** Specific sets of commands determine the parameters to select target-specific gRNAs. Target sites are composed of a variable sequence (the “guide sequence”) adjacent to a specific sequence motif (the “PAM sequence”). The help documentation and Vignettes mentioned above describe these commands in detail. To start, try the command sets described here and modify the types of sites identified (e.g. only paired sites, TRUE or FALSE) and the species used for off-target analysis.

*A. Run “offTargetAnalysis” from CRISPRseek.* Make sure your input fasta file is in your working directory (Step 4). Then, paste the following command set into the R console. You need to change some or all of the text in bold, including your filename (**X**), whether to restrict only to sites that are in pairs or overlap with a restriction site (**TRUE** or **FALSE**) and how many mismatches to allow in your off-target search. For an initial survey of candidate sites without the more time-consuming off-target analysis, leave *chromToSearch* empty.

*library(CRISPRseek)*

*library("BSgenome.Hsapiens.UCSC.hg19")*

*library(TxDb.Hsapiens.UCSC.hg19.knownGene)*

*outputDir <- getwd()*

*inputFilePath <- "****X****.fa"*

*REpatternFile <- system.file("extdata", "NEBenzymes.fa", package = "CRISPRseek")*

*offTargetAnalysis(inputFilePath, findgRNAsWithREcutOnly =* ***FALSE****,*

*REpatternFile = REpatternFile, findPairedgRNAOnly =* ***FALSE****, BSgenomeName = Hsapiens, txdb = TxDb.Hsapiens.UCSC.hg19.knownGene, max.mismatch =* ***3****, chromToSearch = "", outputDir = outputDir, overwrite = TRUE)*

The output files will be in the working directory. To perform a genome wide off-target search, use *chromToSearch = "****all***". We use the combined off-target scores of the 5 or 10 most likely off-target sites to select gRNAs with a lower probability of off-target cutting. gRNAs that overlap restriction sites (REs) or are in paired configurations may also be selected.

*B. Perform off-target analysis with a different species.* Below is an example of a similar script, but using the mouse genome for off-target analysis; the mouse genome and annotation packages must be installed first.

*library(CRISPRseek)*

*library("****BSgenome.Mmusculus.UCSC.mm10****")*

*library(****TxDb.Mmusculus.UCSC.mm10.knownGene****)*

*outputDir <- getwd()*

*inputFilePath <- "****X****.fa"*

*REpatternFile <- system.file("extdata", "NEBenzymes.fa", package = "CRISPRseek")*

*offTargetAnalysis(inputFilePath, findgRNAsWithREcutOnly = FALSE,*

*REpatternFile = REpatternFile, findPairedgRNAOnly = FALSE, BSgenomeName =* ***Mmusculus,*** *txdb =* ***TxDb.Mmusculus.UCSC.mm10.knownGene****, max.mismatch = 3, chromToSearch = "****all****", outputDir = outputDir, overwrite = TRUE)*

*C. Perform off-target analysis on an existing set of candidate gRNAs.* If you would like to perform the off-target analysis on an existing set of candidate gRNAs, you can start with a fasta format file with each of the target sites (e.g. the gRNA plus PAM sequence). Set *findgRNAs =* **FALSE** to bypass the step of identifying gRNAs and proceed to an off-target analysis of the gRNAs in the input sequence.

*library(CRISPRseek)*

*library("BSgenome.Hsapiens.UCSC.hg19")*

*library(TxDb.Hsapiens.UCSC.hg19.knownGene)*

*outputDir <- getwd()*

*inputFilePath <- "****X****.fa"*

*REpatternFile <- system.file("extdata", "NEBenzymes.fa", package = "CRISPRseek")*

*offTargetAnalysis(inputFilePath, findgRNAs* = **FALSE**, *findgRNAsWithREcutOnly =* ***FALSE****, REpatternFile = REpatternFile, findPairedgRNAOnly =* ***FALSE****, BSgenomeName = Hsapiens, txdb = TxDb.Hsapiens.UCSC.hg19.knownGene, max.mismatch =* ***3****, chromToSearch = "****all****", outputDir = outputDir, overwrite = TRUE)*

*D. Rank gRNAs based on their ability to act on two different input sequences.* The compare2Sequences function allows you to identify gRNAs for two input sequences.

*library(CRISPRseek)*

*outputDir <- getwd()*

*inputFile1Path <- "****X1****.fa"*

*inputFile2Path <- "****X2****.fa"*

*REpatternFile <- system.file("extdata", "NEBenzymes.fa", package = "CRISPRseek")*

*seqs <- compare2Sequences(inputFile1Path, inputFile2Path, outputDir = outputDir , REpatternFile = REpatternFile, overwrite = TRUE)*

*seqs*

***X1***.fa and ***X2***.fa are the names of the two input fasta files. The output file will list all of the possible gRNAs for each of the two input sequences and provide a cleavage score for each gRNA for each of the two input sequences. Selected gRNAs can then be examined for off-target sequences as described in Step 5C**.**
